# PMN-PT/PVDF Nanocomposite for High Output Nanogenerator Applications

**DOI:** 10.3390/nano6040067

**Published:** 2016-04-11

**Authors:** Chuan Li, Wenbo Luo, Xingzhao Liu, Dong Xu, Kai He

**Affiliations:** 1 State Key Laboratory of Electronic Thin Film and Integrated Devices, School of Microelectronics and Solid-State Electronics, University of Electronic Science and Technology of China, Chengdu 610054, China; uestc_lich@hotmail.com (C.L.); xzliu@uestc.edu.cn (X.L.); frank@shu.edu.cn (D.X.); 2 School of Material Science and Engineering, Jiangsu University, Zhenjiang 212013, China; hekai40@126.com

**Keywords:** (1−*x*)Pb(Mg_1/3_Nb_2/3_)O_3_-*x*PbTiO_3_ (PMN-PT), poly(vinylidene fluoride) (PVDF), piezoelectric, nanocomposites, tape-casting, flexible, nanogenerator

## Abstract

The 0.7Pb(Mg_1/3_Nb_2/3_)O_3_-0.3PbTiO_3_(0.7PMN-0.3PT) nanorods were obtained via hydrothermal method with high yield (over 78%). Then, new piezoelectric nanocomposites based on (1−*x*)Pb(Mg_1/3_Nb_2/3_)O_3_-*x*PbTiO_3_ (PMN-PT) nanorods were fabricated by dispersing the 0.7PMN-0.3PT nanorods into piezoelectric poly(vinylidene fluoride) (PVDF) polymer. The mechanical behaviors of the nanocomposites were investigated. The voltage and current generation of PMN-PT/PVDF nanocomposites were also measured. The results showed that the tensile strength, yield strength, and Young’s modulus of nanocomposites were enhanced as compared to that of the pure PVDF. The largest Young’s modulus of 1.71 GPa was found in the samples with 20 wt % nanorod content. The maximum output voltage of 10.3 V and output current of 46 nA were obtained in the samples with 20 wt % nanorod content, which was able to provide a 13-fold larger output voltage and a 4.5-fold larger output current than that of pure PVDF piezoelectric polymer. The current density of PMN-PT/PVDF nanocomposites is 20 nA/cm^2^. The PMN-PT/PVDF nanocomposites exhibited great potential for flexible self-powered sensing applications.

## 1. Introduction

Ferroelectric materials have brilliant dielectric, ferroelectric, piezoelectric, pyroelectric, and nonlinear optic properties could be used in microwave devices, non-random access memories, energy conversion devices, and sensors [1,2]. The current state-of-the-art technologies suffer from low energy density, making them bulky and costly. Piezoelectric nanostructures provide a practical way to harvest energy from the environment to power nanodevices and nanosystems [3,4,5]. They can also be used as novel self-powered sensing devices. Recently, the piezoelectric nanocomposites, which are produced by dispersing piezoelectric nanoparticles in a flexible matrix, has attracted much attention. Park *et al* [6] developed a nanocomposite generator by dispersing BaTiO_3_ nanoparticles and graphitic carbon mixture in polydimethylsiloxane matrix, which generated an output voltage of 3.2 V and a current of 350 nA. Jung *et al* developed a NaNbO_3_ nanowire-polydimethylsiloxane (PDMS) polymer nanocomposite device, which provides an output voltage of 3.2 V and an output current of 72 nA, could drive small electronic devices [7].

One efficient way to increase the output voltage of those piezoelectric nanodevices is a synthesis of the piezoelectric materials with a higher piezoelectric constant. Compared with other piezoelectric materials, a solid solution (1−*x*)Pb(Mg_1/3_Nb_2/3_)O_3_-*x*PbTiO_3_ (PMN-PT) compound is well-known as a relaxor ferroelectric compound with a very high dielectric anomaly for the inhomogeneous distribution of B-site cations in the perovskite lattice of Pb(Mg_1/3_Nb_2/3_)O_3_. Moreover, the addition of PbTiO_3_ [8,9,10] is promising for both further research and applications with a high piezoelectric effect of 2500 pC/N [11,12,13]. In addition, Sun *et al* [14] calculated the energy-harvesting properties of the different ZnO, BaTiO_3_, and PMN-PT nanostructure via the vibration of these nanostructures agitated by the ambient vibration energy.

In this letter, a new piezoelectric nanocomposite based on a PMN-PT nanorod was reported. The 0.7PMN-0.3PT nanorods synthesized via a hydrothermal process. These nanorods were mixed with piezoelectric poly(vinylidene fluoride) (PVDF), which prevents the breaking and cracking of embedded piezoelectric nanorods under mechanical stress, to produce a piezoelectric nanocomposite. The nanocomposite was tape-casted onto a metal-coated polyimide substrate and subsequently cured. Voltage and current generation of the PMN-PT nanocomposites were measured during a mechanical tapping. The result shows that PMN-PT nanocomposite produced a high output voltage and current, and thus a high piezoelectric coefficient of both the PMN-PT nanorods and PVDF polymer. The voltage generation obviously increased with the increase in PMN-PT content in the nanocomposite. The PMN-PT/PVDF nanocomposite is a promising material for use in flexible self-powered sensing applications.

## 2. Results and Discussion

**Figure 1:**
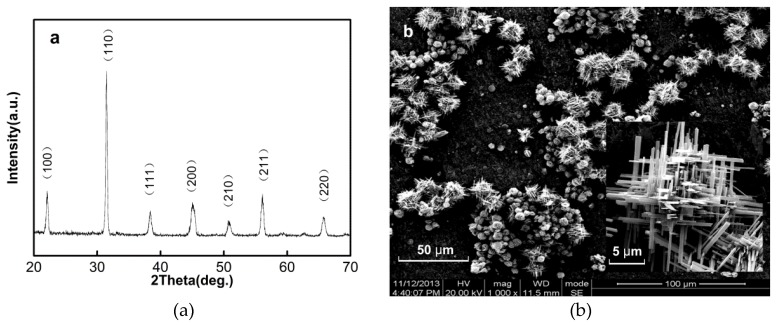
(**a**) X-ray powder diffraction (XRD) pattern of the (1−*x*)Pb(Mg_1/3_Nb_2/3_)O_3_-*x*PbTiO_3_ (PMN-PT) nanorods; (**b**) scanning electron microscope (SEM) images of PMN-PT nanorods, and the inset indicates the corresponding higher magnification SEM image.

Figure 1a shows the X-ray powder diffraction (XRD) pattern of synthesized PMN-PT nanorods. Only the perovskite phase could be observed; no pyrochlore phase is detected by XRD analysis. It indicates that these nanostructures have a well-crystallized perovskite structure with lattice constants of *a* = 0.403 nm, *b* = 0.401 nm, and *c* = 0.402 nm. It is close to the lattice constants value of 0.72PMN-0.28PT nanowire in reference [11]. Figure 1b shows the scanning electron microscope (SEM) image of the PMN-PT nanorod clusters obtained from the hydrothermal synthesis. The size of an individual nanorod clusters ranges from about 15 to 30 μm. The yield of PMN-PT nanorods is over 78%, and the byproduct is in nanoparticle form. The inset high magnification SEM image indicates that the PMN-PT nanorod clusters have a fern-branch three-dimensional structure. There are also some individual nanorods. The length of single nanorod is about 5–10 μm, and the aspect ratio is in a range of 15–30.

**Figure 2:**
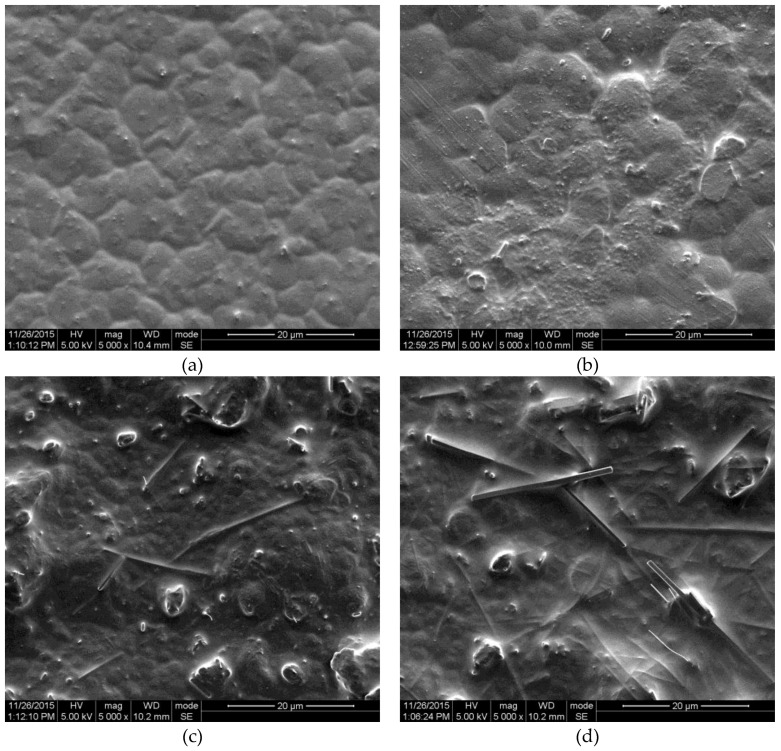
Surface morphology of nanocomposite films with (**a**) 0 wt %; (**b**) 10 wt %; (**c**) 20 wt % and (**d**) 25 wt % PMN-PT nanorod content.

The surface SEM images of the PMN-PT/PVDF nanocomposite with different nanorod content values are shown in Figure 2a–d. The corresponding nanorod content values are 0 wt %, 10 wt %, 20 wt %, and 25 wt %, respectively. Pure PVDF film shows a uniform surface morphology, as shown in Figure 2a. The PMN-PT nanorods are well distributed throughout the PVDF matrix as shown in Figure 2b,c. With the increase in nanorod content, the individual nanorods become close to each other and linked, as shown in Figure 2d. When the composite material is subjected to an external force, the PMN-PT nanorods and PVDF matrix both generate electric potential. Moreover, the flexible PVDF matrix prevents the breaking and cracking of embedded PMN-PT nanorods under mechanical stress.

**Figure 3:**
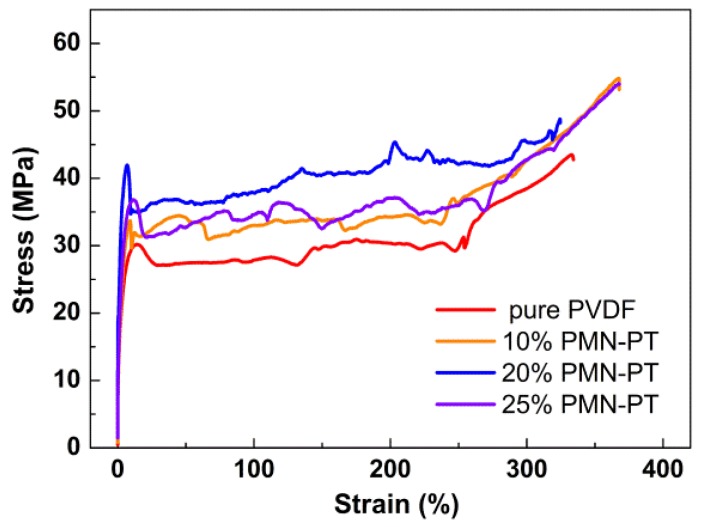
Stress–strain curves of nanocomposites with different PMN-PT content.

The stress–strain curves of nanocomposites with different PMN-PT content are shown in Figure 3. The stress–strain curve of nanocomposites followed the typical stress–strain behavior of polymers. The yield terrace and strain hardening behavior can be clearly seen. Five tensile tests are conducted for each sample, and the measurements are averaged. The average values and standard deviation (σ^2^) are listed in Table 1. The tensile strength (σ_t_), yield strength (σ_s_), and Young’s modulus (*E*) of nanocomposites are enhanced as compared with that of the pure PVDF. The strengthening mechanism of PMN-PT/PVDF composite can be explained as a fiber-reinforced mechanism. The mechanical stress transferred to PMN-PT nanorods through the generation of shear stresses at the nanorod-PVDF interface. The strength and elastic modulus of PMN-PT ceramic is much larger than that of PVDF matrix. Thus, the tensile strength and Young’s modulus of nanocomposites increased with increasing PMN-PT nanorod content. However, the contact between nanorods also increased with nanorod content, which would significantly reduce the stress transfer efficiency at the interface of the nanorod and PVDF. Therefore, the mechanical performance of composites declined when PMN-PT content exceeded 20%. It is worth noting that the Young’s modulus of 20 wt % nanocomposite is 1.71 Gpa, which is 51% larger than that of the pure PVDF sample.

**Figure 4:**
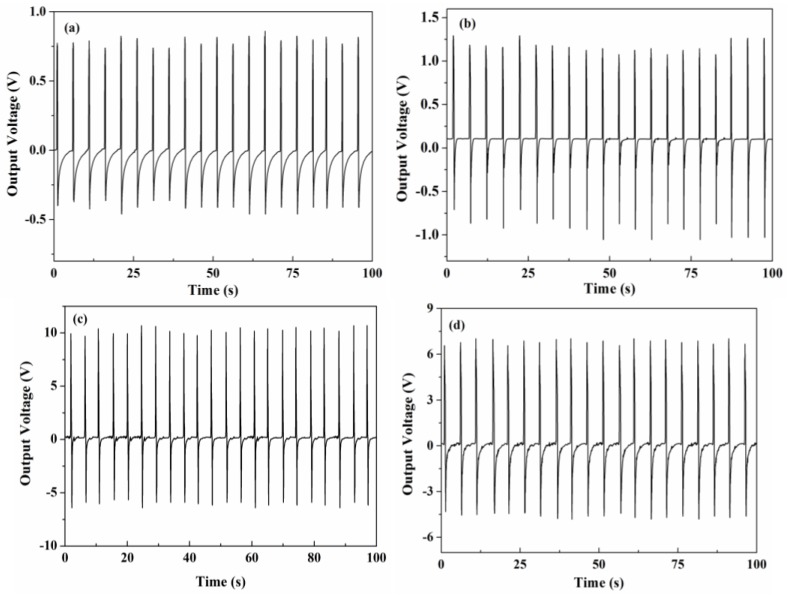
Voltage generation under a periodic mechanical tapping. (**a**) Pure PVDF; (**b**) 10 wt % PMN-PT; (**c**) 20 wt % PMN-PT; and (**d**) 25 wt % PMN-PT.

Voltage generations of the nanocomposites are assessed by tapping the device periodically using a mechanical hammer. The device were tapped by the plastic hammer vertically when a test is triggered manually, and the tapping force is controlled by a spring. It represents the actual application of energy harvesting piezoelectric generators. The voltage generations of the nanocomposites under the same mechanical tapping force shown in Figure 4a–d. It is clear that each tapping generated two voltage peaks. The positive peak corresponds to the direct impact of the stress. The following negative voltage peak corresponds to the damping effect occurring when the device deformation is recovering [15]. The positive peak is greater than the negative peak, because the PMN-PT composite is polarized. The pure PVDF device repeatedly generates voltages ranging from 0.6 to 0.8 V in an open circuit. The generate voltages of nanocomposites significantly increase with the increase in PMN-PT content due to the fact that the piezoelectric performance of nanocomposites are mainly attributed to PMN-PT nanorods. The 20 wt % nanocomposite exhibit the largest generated voltage value (9.8–10.3 V). However, the stress applied on the PMN-PT nanorod is composed of two components: the direct external mechanical stress and shear stresses from the nanorod-PVDF interface. The generate voltage decreased to 6.3–6.7 V when nanorod content increased to 25 wt %, which, due to the deformation of nanorods, decreased with the increase in nanorod content as a consequence of the bad contact at the nanorod-PVDF interface. The maximum generate voltage of PMN-PT nanocomposite is three times larger than that of the nanocomposite based on BaTiO_3_ and NaNbO_3_ (3.2 V) [6,7]. It is also larger than the PMN-PT/PDMS nanocomposite ranging from 4.2 to 7.8 V [15].

**Figure 5:**
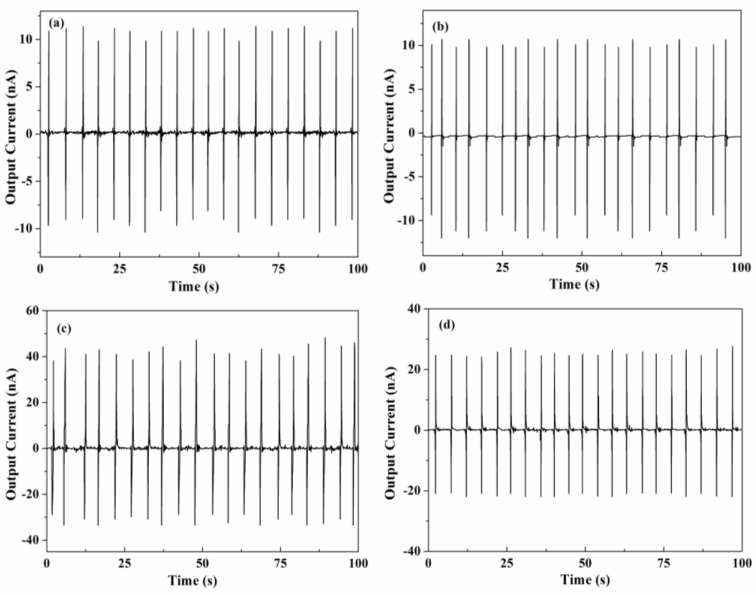
Current generation under a periodic mechanical tapping. (**a**) Pure PVDF; (**b**) 10 wt % PMN-PT; (**c**) 20 wt % PMN-PT; and (**d**) 25% PMN-PT.

Current generations of the nanocomposites are shown in Figure 5a–d. Pure PVDF has the smallest current value of 9.8–10.4 nA. 10 wt % nanocomposites have the same generated current as pure PVDF due to the low PMN-PT content. 20 wt % nanocomposites have the largest generate current value of 38–46 nA. The current density of PMN-PT/PVDF nanocomposites is about 20 nA/cm^2^, which is higher than that of BaTiO_3_ and NaNbO_3_ nanocomposites ranging from 5 to 16 nA/cm^2^ [6,7]. The difference between the positive and negative current peaks is not obvious. The generated current depends on the strength of the piezoelectric potential. Theoretically, the piezoelectric potentials are generated by the direct impact, and the relaxed stress makes no significant difference [15]. The nanocomposite cannot be simply treated as a homogeneous system. Young’s modulus of piezoelectric ceramic is significantly larger than that of polymer. The slight difference between generated currents can be attributed to the variation of volume resistivity during the deformation process.

When mechanical stress is applied to the piezoelectric composite, the stress is transferred through the polymer matrix and piezoelectric nanowire. The electric potential gradient is generated by mechanical stress, which can be transferred to electrodes to form a piezoelectric potential. Moreover, it can also be applied to an external circuit. The output voltage (*V*_out_) can be calculated by the following equation:

(1) $$V_{out}=\int g_{33}\varepsilon \left(l\right)Edl$$

where *g*_33_ is the piezoelectric voltage constant, ε(*l*) is the strain perpendicular to the electrodes, *E* is Young’s modulus, and *dl* is the integration of nanorods perpendicular to the electrodes. The large piezoelectric coupling coefficient *d*_33_ value of the 0.7PMN-0.3PT nanorod (409 pm/V) [16] and the piezoelectric PVDF matrix provide a large *g*_33_ value. However, the Young’s modulus of the PMN-PT nanorod is much larger than that of the PVDF matrix. With the PMN-PT nanorods randomly dispersed in the polymer matrix, a direct mechanical impact cannot be transferred to a nanorod effectively. Nanorods perpendicular to the electrodes would have the best performance to enhance the effciency of the mechanical impact transfer, which corresponds to a large ε(*l*) and dl. Otherwise, the dielectric constant of piezoelectric composite is much lower than that of the corresponding pure piezoelectric material [17]. Therefore, the significant performance of output voltage and current density are attributed to both the higher piezoelectric constant and the unique piezoelectric nanostructures.

## 3. Experimental Section

### 3.1. Chemicals

Lead (II) acetate trihydrate, niobium (V) ethoxide, titanium diisopropoxide bisacetyl acetonate (TIAA), dimethylformamide (DMF), and polyacrylic acid (PAA) were purchased from Alfa Aesar (Shanghai, China). Magnesium 2,4-pentanedionante dehydrate (MgAA) and 1, 1, 1-tris (methylol)ethane (THOME) were purchased from Aike reagent (Chengdu, China). Poly (ethylene glycol)-200 (PEG200), methanol (MeOH), and KOH were purchased from Kelong chemical reagent (Chengdu, China). Poly(vinylidene fluoride) was purchased from HWKR Chem Co. Ltd (Beijing, China). Deionized (DI) water was used throughout the experiment.

### 3.2. Preparation of 0.7PMN-0.3PT Nanorods

The synthesis of 0.7PMN-0.3PT nanorods was performed via the hydrothermal method [18]. Stoichiometric amounts (corresponding to the 0.7PMN-0.3PT composition) of lead (II) acetate trihydrate (0.3793 g), MgAA (0.0603 g), niobium ethoxide (0.12 mL), and TIAA (0.11 mL) were mixed in PEG200 and MeOH mixture (PEG200/MeOH at a 1:2 volume ratio) using THOME (0.12 g) as the complexing agent to form a Pb–Mg–Nb–Ti (PMNT) sol-gel. The concentration of the PMNT sol-gel was 0.01 M. Six milliliters of PMNT sol-gel were dispersed into DI water to form a yellow solution with a strong stirring in the ratio of 1:10. After that, 0.05 mL of PAA and 20 g of KOH were added into the yellow solution with quickly stirring, and a white precipitate was formed. The suspension was sealed into a Teflon-lined stainless steel autoclave (80 mL in volume) and kept in an oven at 235 °C for 24 h. After cooling to room temperature, the suspension was washed with ethanol and DI water 6 times and dried at 100 °C in an oven for 6 h. Grey powder consisting of (100) oriented PMN-PT nanorods were obtained [16]. The yield of PMN-PT nanorods was over 78%.

### 3.3. Preparation of PMN-PT/PVDF Composite and Nanogenerator

The PVDF powder was pre-dissolved in DMF in a ratio of 10:1 *w*/*w*. Then, PMN-PT nanorods were weighted (0 g, 0.556 g, 1.25 g, and 1.667 g) and added into 5 g of a PVDF solution with a mechanical mixing, and the corresponding PMN-PT content were 0 wt %, 10 wt %, 20 wt %, and 25 wt %, respectively. The well-mixed slurry was tape-casted onto a glass plate, and DMF was allowed to evaporate slowly. After curing at 80 °C for 10 min on a hotplate, the nanocomposite film was peeled off from the glass substrate and cured at 120 °C for 2 h with uniform pressure in vacuum condition. A flat and uniform film of PMN-PT/PVDF composite with a thickness of 150 μm was obtained. The composite films were cut into 1.5 cm × 1.5 cm square pieces and plated with Ni (50 nm)/Au (150 nm) electrodes on both sides of the pieces. Then, the nanogenerators were poled with an electric field of 5 kV/mm at 110 °C in a silicone oil bath for 4 h.

### 3.4. Characterization

The morphology characteristics of nanorods and nanocomposites were characterized with a scanning electron microscope (SEM, Inspect F50, FEI, Hillsboro, OR, USA). The structure of the PMN-PT nanorod was examined using a powder X-ray diffractometer (XRD, DX1000, Tongda Sicence and Technology Co. Ltd, Dandong, China) with CuKα of 0.1542-nm (40 kV, 30 mA) radiation. The mechanical behavior was investigated by performing a tensile test using the Instron 5567 material testing system (Norwood, MA, USA). Voltage and current generation of the nanogenerators were measured during a mechanical tapping using an Agilent 4155C semiconductor parameter analyzer (Santa Clara, CA, USA).

## 4. Conclusions

The PMN-PT nanorods were obtained by hydrothermal reaction. Then, a new piezoelectric nanocomposite based on the PMN-PT nanorod was fabricated by dispersing the nanorods into the piezoelectric PVDF polymer. The PMN-PT nanorods were well distributed throughout the PVDF matrix. The tensile strength, yield strength, and Young’s modulus of nanocomposites were enhanced, as compared with that of the pure PVDF. The Young’s modulus first increased and then decreased with increasing nanorod content, and the largest Young’s modulus was obtained at 20 wt % nanorod content. The nanogenerators were fabricated with the PMN-PT/PVDF nanocomposites. The maximum output voltage of 10.3 V and output current of 46 nA were obtained when PMN-PT nanorod content was 20 wt %, which provided a 13-fold larger output voltage and 4.5-fold larger output current than that of the pure PVDF piezoelectric polymer. The maximum generated voltage of the PMN-PT nanocomposite was three times larger than that of nanocomposite based on BaTiO_3_ and NaNbO_3_. It was also larger than the PMN-PT/PDMS nanocomposite ranging from 4.2 to 7.8 V. The current density of PMN-PT/PVDF nanocomposites is 20 nA/cm^2^, which is much higher than that of BaTiO_3_ and NaNbO_3_ nanocomposites. The excellent performance of output voltage and current density is attributed to both the higher piezoelectric constant and the unique piezoelectric nanostructures. PMN-PT/PVDF nanocomposites are promising materials for energy harvesting and self-powered sensing applications.

## Figures and Tables

**Table 1:** Tensile testing results of PMN-PT/poly(vinylidene fluoride) (PVDF) nanocomposites.

PMN-PT Content (wt %)	Tensile Strength (MPa)		Yield Strength (MPa)		Young’s Modulus (GPa)	
	$\bar{\sigma_{t}}$	σ^2^	$\bar{\sigma_{s}}$	σ^2^	$\bar{\sigma_{t}}$	σ^2^
0	43.48	0.84	26.01	0.42	1.13	0.15
10	54.84	1.22	31.41	0.53	1.47	0.17
20	48.78	1.17	30.74	0.46	1.71	0.13
25	44.87	1.06	31.47	0.41	1.25	0.14

## Abbreviations

The following abbreviations are used in this manuscript:

PAA	polyacrylic acid
PDMS	polydimethylsiloxane
PMN-PT	(1−*x*)Pb(Mg_1/3_Nb_2/3_)O_3_-*x*PbTiO_3_
PVDF	poly(vinylidene fluoride)
XRD	X-ray diffraction
SEM	scanning electron microscopy
TIAA	titanium diisopropoxide bisacetyl acetonate
DMF	dimethylformamide
PAA	polyacrylic acid
MgAA	magnesium 2,4-pentanedionante dehydrate
THOME	1, 1, 1-tris (methylol) ethane
PEG200	poly(ethylene glycol)-200
MeOH	methanol
DI	deionized
PMNT	Pb–Mg–Nb–Ti
